# Correlations between iodine status and the risk of thyroid nodules, a systematic review and dose–response meta-analysis

**DOI:** 10.3389/fendo.2026.1711749

**Published:** 2026-01-27

**Authors:** Changbo Lu, He Dong, Peishen Shi, Weichen Dong, Xinxin Wen, Qiuming Gao

**Affiliations:** 1 Department of Orthopedics, 940th Hospital, Lanzhou, Gansu, China; 2 Departments of Orthopaedic Surgery, Xijing Hospital, Air Force Medical University, Xi’an, Shaanxi, China; 3 Department of Thyroid, Breast and Vascular Surgery, Xijing Hospital, Air Force Medical University, Xi’an, Shaanxi, China

**Keywords:** dose-response analysis, iodine intake, risk factors, thyroid nodules, urinary iodine concentration

## Abstract

**Background:**

The global incidence of thyroid nodules is rising substantially. The correlation between excessive iodine intake and an elevated risk of thyroid nodules remains a subject of debate.

**Objective:**

To evaluate the categorical and quantitative dose-response associations between iodine status levels and the risk of thyroid nodules.

**Methods:**

We systematically searched PubMed, Web of science, Embase, Cochrane Library and Scopus for studies published before December 2025 that investigated the association between iodine status and thyroid nodules, without language restrictions. The categorical association was assessed by pooling odds ratios (ORs) for thyroid nodules across different iodine status categories, using the adequate category as the reference. The continuous dose-response association was evaluated using random-effects generalized least squares spline models. The primary outcome was the prevalence of thyroid nodules at different iodine status statuses.

**Results:**

This meta-analysis included 25 cross-sectional studies comprising 54621 participants (57.7% women) and 13569 thyroid nodule events. In categorical analyses, participants in the iodine deficiency (median urinary iodine concentration [UIC]: 50 μg/L) showed higher odds of thyroid nodules (OR = 1.28, 95%CI=1.09-1.50) compared to the adequate iodine category (median UIC, 150 μg/L). No significant association was found for both the more-than-adequate iodine (median UIC, 250 μg/L) category and excessive iodine (median UIC, 350 μg/L) categories (OR = 1.02, 95% CI = 0.93-1.11; OR = 1.13, 95%CI=0.98-1.30, respectively). Nine studies reporting continuous UIC outcomes, the mean difference (MD) between participants with and without nodules was 4.11 (95% CI, 2.51 to 5.71, P < 0.01). Continuous dose-response analysis revealed a significant U-shaped nonlinear correlation (P for nonlinearity < 0.001), with increased risks at both deficient and excessive iodine levels. These associations remained consistent in analyses by unadjusted variables of sex, age and BMI. Further analysis stratified by geographical factors also revealed similar correlation tendency.

**Conclusions:**

The relationship between iodine intake and thyroid nodule risk follows a U-shaped, nonlinear pattern, where both deficiency and excess are associated with increased risks. More than adequate iodine intake showed a trend toward a lower prevalence of thyroid nodules. However, this observation should be interpreted with caution due to wide confidence intervals, heterogeneity, and potential residual confounding.

**Systematic Review Registration:**

PROSPERO, https://www.crd.york.ac.uk/PROSPERO/view/CRD420251236621, identifier CRD420251236621.

## Introduction

The prevalence of thyroid nodules was increasing sharply in recent years, with a range of 4% to 7% observed through physical examination and 19% to 67% through when using sensitive imaging techniques such as ultrasound (1–3). The dramatic rise in asymptomatic nodules can be partially ascribed to the growing diagnostic scrutiny and imaging applications (4–9). Given that thyroid nodularity can cause thyroid dysfunction, such as hyperthyroidism, and that around 10% of thyroid nodules are prone to malignancy (10), identifying its modifiable risk factors is a public health priority. Established risk factors include age, female gender, and family history, but the role of iodine status status remains particularly controversial.

Iodine is an essential micronutrient for the synthesis of thyroid hormones. Historically, iodine deficiency has been a well-established cause of thyroid goiter and nodules, primarily through mechanisms involving TSH-stimulated follicular cell hyperplasia and oxidative stress-induced damage (11, 12). To address iodine deficiency disorders, China implemented a Universal Salt Iodization (USI) policy in 1996. While this policy successfully reduced the rates of goiter, the national prevalence of thyroid nodules has paradoxically risen sharply, from 2.73% in 1999 to 20.43% in 2017 (13, 14). This temporal correlation has sparked concern and debate over whether excessive iodine intake, resulting from USI, could be a contributing factor.

However, the epidemiological evidence linking high iodine intake to thyroid nodule risk is inconsistent (12, 15–17). Several studies have reported a higher prevalence of thyroid nodules in populations with excessive iodine status compared to those with adequate levels (15, 18–23). In contrast, other research has found no significant association (24, 25) or even suggested a protective effect of more-than-adequate iodine intake. Additionally, some evidence points to a U-shaped relationship, where both deficiency and excess of iodine are associated with an elevated risk of thyroid nodules (15, 19–21). This ongoing controversy highlights a critical knowledge gap, the absence of a definitive, quantitative dose-response relationship between iodine status levels and thyroid nodule risk.

To address this critical knowledge gap, we conducted the present systematic review and meta-analysis. Our primary objective was to quantitatively synthesize the epidemiological evidence to establish a definitive dose-response relationship between iodine status, measured by urinary iodine concentration (UIC), and the risk of thyroid nodules. This study provides novel insights by employing both categorical and continuous modeling approaches to delineate the precise nature of this association, moving beyond the inconsistent findings of prior research to inform a more nuanced understanding of iodine-related thyroid nodule risk.

## Materials and methods

Our review was prospectively registered in PROSPERO (CRD420251236621) and followed Meta-Analyses of Observational Studies in Epidemiology (MOOSE) reporting guidelines (26).

### Search strategy

A systematic search was performed in the databases of PubMed, Web of science, Embase, Cochrane Library and Scopus without language restrictions, from inception to December, 2025. The search aimed to extract the studies reporting population-based data on the prevalence of thyroid nodules, odds ratios (ORs) comparing thyroid nodule risk across iodine categories, and corresponding iodine intake levels measured as UIC values. The search strategy was designed to encompass three core concepts: (1) iodine status, (2) thyroid nodules, and (3) epidemiological study designs. The systematically developed search strategy was based on the PICO(S) scheme. Due to the research question, only keywords/MESHs in the categories population, intervention and outcome were considered. The full search strategy is available in the **Supplementary Materials**.

### Study selection

The inclusion criteria were as follows: (1) Population: Adults from general community or outpatient settings. The study population for the control or reference group was required to be explicitly described as healthy, euthyroid, and representative of the local resident population; (2) Exposure/Indicator: iodine statusal status, quantitatively measured using UIC; (3) Comparator: different levels of iodine status (e.g., deficient, sufficient, excessive) as defined by the study authors or WHO criteria; (4) Outcome: the prevalence or odds of thyroid nodules, diagnosed by thyroid ultrasonography; (5) Study Design: observational studies, including cross-sectional, case-control, or cohort studies, with a minimum sample size of 20 participants to ensure statistical reliability.

**Figure 1:**
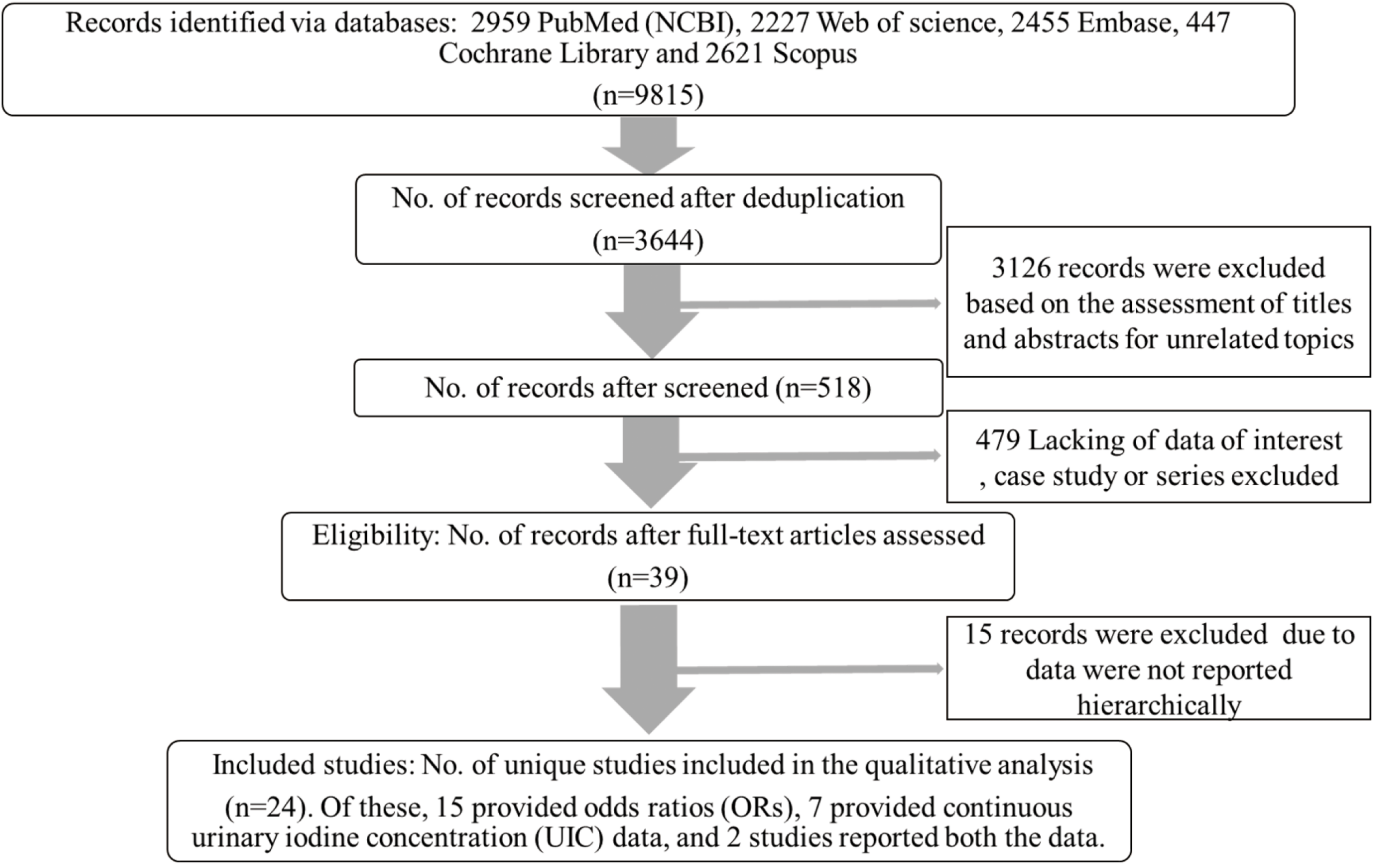
PRISMA flow diagram of study selection.

The exclusion criteria were as follows: (1) reviews, editorial or case reports; (2) studies lacking major data, like iodine status status measured by UIC; (3) studies including participants afflicted with any related diseases or took medicines known to affect thyroid structure or function and (4) the studies focused on participants with one underlying condition (studies focused on participants taking medications known to significantly affect thyroid structure or function (e.g., lithium, amiodarone, thyroid hormone replacement therapy). All retrieved articles were carefully examined, and any citations that were overlooked during the search process were scrutinized carefully (**Figure 1**).

### Data extraction and quality assessment

Data from each study reporting median UIC is recognized as the epidemiologic criteria for assessing iodine status status and were initially extracted. The iodine statusal status was identified in accordance with WHO criteria by the median UIC level (27): iodine-inadequate status with MUIC<100 μg/L, iodine-sufficient status with MUIC of 100–199 μg/L, iodine-more than adequate status with MUI of 200–299 μg/L, and a MUIC >300 μg/L to be iodine excessive. The data that was extracted encompassed the following items: study design, year of publication, demographic information of participants, sample size, and incidence of thyroid nodules at each iodine statusal status. The primary outcome was the OR for the prevalence of thyroid nodules, comparing different categories of iodine status. A secondary analysis involved calculating the mean difference (MD) in continuous UIC levels between participants with and without thyroid nodules. To clarify the potential heterogeneity involving demographic and anthropometric variables, effect estimates from the most adjusted model (age, gender and body mass index (BMI)) were conducted. Further dose-response analysis was conducted with geographical factors stratified (coastal versus inland) to explore the influence of geographical differences on the outcomes. Two reviewers independently screened studies and extracted data. Any discrepancies were resolved through discussion or, if necessary, by consultation with a third senior reviewer.

The 11-item checklist recommended by Agency for Healthcare Research and Quality (AHRQ) was employed to assess the methodological quality of the studies included. An item would be scored ‘0’ if it was answered ‘NO’ or ‘UNCLEAR’; if it was answered ‘YES’, then the item scored ‘1’. The evaluation of article quality was conducted in the following manner: low quality = 0– 3; moderate quality = 4–7; high quality = 8–11. The quality scores were used to inform a sensitivity analysis, where analyses were repeated after excluding studies deemed to be of low quality.

The certainty (quality) of the overall body of evidence for the primary dose-response association was assessed using the GRADE (Grading of Recommendations, Assessment, Development, and Evaluations) framework. The initial certainty for observational evidence was rated as ‘Low,’ which was then potentially downgraded or upgraded based on five domains: risk of bias (study limitations), inconsistency (heterogeneity), indirectness, imprecision, and publication bias (**Supplementary Table S1**).

### Data analysis

The statistical analysis was conducted using the R V.4.4.0 and the *dosresmeta* package. Study characteristics were identified using descriptive statistics, such as mean and ranges of the pooled data. Effect estimates were pooled using either a fixed-effects or random-effects model, depending on the heterogeneity analysis, and forest plots were generated to visualize between-group comparisons. A one-stage hierarchical regression model was used for meta-analysis of nonlinear dose-response data. To evaluate publication bias, we analyzed a funnel plot graph to determine its asymmetry when we had more than ten studies. The presence of asymmetry in the funnel plot predicts publication bias.

### Estimation of population risk analysis

Pooled odds ratios (OR) with 95% confidence intervals (CI) for the risk of high iodine intake was assessed. We calculated unadjusted or adjusted (adjustment for BMI, gender and age) ORs for the overall effect estimate. A control group consisting of individuals with an adequate iodine status (interquartile range, 100-199 μg/L) was established. The median doses of the pooled excessive, more-than-adequate, and inadequate categories were 350 (interquartile range, 100-199 μg/L), 250 (interquartile range, 100-199 μg/L), and 50 (interquartile range, 100-199 μg/L) ug/L, respectively. Mean differences (MD) with 95% CI were used to assess the effect size for continuous data. Pooled analysis comparing excessive/inadequate with adequate iodine intake included all 17 available studies, and 16 studies comparing more than adequate versus adequate iodine status categories. Potential heterogeneity among studies was evaluated by the Higgins’ *I*^2^ test, and heterogeneity was statistically assessed using the *Q* statistic (28). All *P* values were-2 tailed. A probability level below 0.05 was considered statistically significant for all tests.

### Continuous dose-response analysis

Nonlinearity in the association between iodine status and thyroid nodules risk was assessed by modeling UIC by fitting restricted cubic spline model with 3/4 knots at fixed centiles (5%, 50%, and 95%/5th, 35th, 65th and 95th) of the distribution. Initially, we first estimated a restricted cubic spline model using a generalized least squares regression, taking into accounts the correlation within each set of reported ORs. Subsequently, we combined the study-specific estimates, employing the restricted maximum likelihood approach in a multivariable random-effects meta-analysis. The robusthyroid noduleess of the observed associations was assessed through conducting subgroup analyses based on geographic location (coastal vs inland studies). The rationale for this classification is that populations residing in coastal regions typically have a diet richer in various types of seafood and marine products, leading to more diverse sources and generally higher levels of iodine intake compared to inland populations. Additionally, we conducted sensitivity analyses utilizing odds ratios from models that did not account for physical activity and BMI levels, aiming to ascertain if the observed correlation between dosage and response differed between those with and without accounting for these potential confounding factors.

## Results

The study selection process is detailed in **Figure 1**. The initial literature search identified 3644 records after removing duplicates. 3126 records were excluded based on title and abstract screening. A further 479 studies were excluded for lacking primary data of interest, and 15 were excluded due to the absence of hierarchical UIC data. Ultimately, 24 articles met the inclusion criteria (12, 15, 18–22, 29–45). Of these, 15 provided odds ratios (ORs), 7 provided continuous urinary iodine concentration (UIC) data, and 2 study reported both types of data. The key characteristics of the included studies are presented in **Table 1** and **Table 2**.

**Table 1:** Iodine status categories and associated risk for thyroid nodules across included studies.

Source	Geography	Iodine status categories	Most adjusted OR by category (95%CI)	Covariates in the most adjusted model
Yu (29) et al., 2021	Inland	Urine iodine concentration (UIC), μg/L 1. <100 μg/L 2.100–199μg/L (reference) 3. 200–299 μg/L 4. >300 μg/L	1:0.62 (0.31-1.22) 2:1 (reference) 3:0.86 (0.57-1.29) 4:1.15 (0.7-1.89)	Unadjusted
Zhao (30) et al., 2014	Coastal	Urine iodine concentration (UIC), μg/L 1. <100 μg/L 2.100–199μg/L (reference) 3. 200–299 μg/L 4. >300 μg/L	1:0.93 (0.77-1.12) 2:1 (reference) 3:1.22 (1.04-1.43) 4:1.24 (1.04-1.48)	Adjusted for age, TSH, TPOAb, TGAb, and nodule number
Chen (12)et al., 2013	Coastal	Urine iodine concentration (UIC), μg/L 1. <100 μg/L 2.100–199μg/L (reference) 3. 200–299 μg/L 4. >300 μg/L	1:1.25 (1.07-1.45) 2:1 (reference) 3:1.01 (0.86-1.18) 4:0.97 (0.82-1.14)	Adjusted for age, sex, BMI, education, marriage, cigarette smoking
Di-en Yan (21) et al., 2023	Coastal	Urine iodine concentration (UIC), μg/L 1. <100 μg/L 2.100–199μg/L (reference) 3. 200–299 μg/L 4. >300 μg/L	Unadjusted 1:0.9 (0.55-1.73) 2:1 (reference) 3:1.59 (0.92-2.75) 4:3.33 (1.32-8.42) Adjusted1 1:1.22 (0.67–2.20) 2:1 (reference) 3:1.27 (0.73–2.24) 4:3.27 (1.03–6.75) Adjusted2 1:0.97 (0.54–1.77) 2:1 (reference) 3:1.51 (0.88–2.67) 4:3.21 (1.24–8.02)	Adjusted1 for gender- and age; Adjusted2 for SP-, DP-, WC-, HR-, and BMI
Yan (41) et al., 2021	Coastal	Urine iodine concentration (UIC), μg/L 1. <100 μg/L 2.100–199μg/L (reference) 3. 200–299 μg/L 4. >300 μg/L	Rural resident 1:1.35 (0.93-1.95) 2:1 (reference) 3:1.70 (1.17-2.48) 4:1.2 (0.80-1.80) Urban resident 1:1.52 (0.84-2.73) 2:1 (reference) 3:1.63 (0.90-2.95) 4:1.33 (0.70-2.53)	Adjusted for (urban area and rural area), sex, age (continuous variable), smoking status
Gao (32) et al., 2019	Coastal	Urine iodine concentration (UIC), μg/L 1. <100 μg/L 2.100–199μg/L (reference) 3. 200–299 μg/L 4. >300 μg/L	1:1.15 (0.83-1.6) 2:1 (reference) 3:1.03 (0.99-1.02) 4:1.51 (0.96-2.36)	Adjusted for age, BMI, parity, TSH, regions, secondhand smoking, prepregnancy, TPOAb, and TgAb
Zhao (34) et al., 2015	Coastal	Urine iodine concentration (UIC), μg/L 1. <100 μg/L 2.100–199μg/L (reference) 3. 200–299 μg/L 4. >300 μg/L	1:2.19 (1.56-3.07) 2:1 (reference) 3:1.28 (0.70-2.35) 4:2.03 (1.05-3.91)	Unadjusted
Hu (36) et al., 2015	Inland	Urine iodine concentration (UIC), μg/L 1. <100 μg/L 2.100–199μg/L (reference) 3. 200–299 μg/L 4. >300 μg/L	1:1.04 (0.76-1.43) 2:1 (reference) 3:0.97 (0.73-1.30) 4:1.28 (0.94-1.75)	Unadjusted
Lou (20) et al., 2020	Coastal	Urine iodine concentration (UIC), μg/L 1. <100 μg/L 2.100–199μg/L (reference) 3. 200–299 μg/L 4. 300-399 μg/L 5. >300 μg/	1:1.62 (1.27-2.07) 2:1 (reference) 3:0.66 (0.49-0.9) 4:0.62 (0.42-0.93) 5:0.88 (0.47–1.67)	Adjusted for gender, age, education, and profession, BMI, waist circumference
Fan (19) et al., 2017	Coastal	Urine iodine concentration (UIC), μg/L 1. <100 μg/L 2.100–199μg/L (reference) 3.200–299 μg/L 4. >300 μg/L	1:1.62 (1.27-2.07) 2:1 (reference) 3:0.66 (0.49-0.9) 4:0.62 (0.42-0.93)	Unadjusted
Du (15) et al., 2014	Inland	Urine iodine concentration (UIC), μg/L 1. <100 μg/L 2.100–299μg/L (reference) 3. >300 μg/L	1:2.97 (2.05-4.32) 2:1 (reference) 3:1.87 (1.30-2.68)	Unadjusted
Deng (33) et al., 2010	Coastal	Urine iodine concentration (UIC), μg/L 1. <100 μg/L 2.100–199μg/L (reference) 3. 200–299 μg/L 4. >300 μg/L	1:1.44 (1.29-1.61) 2:1 (reference) 3:0.87 (0.78-0.98) 4:0.91 (0.81-1.02)	Unadjusted
Fan (35) et al., 2023	Inland	Urine iodine concentration (UIC), μg/L 1. <100 μg/L 2.100–199μg/L (reference) 3. 200–299 μg/L 4. >300 μg/L	1:1.05 (0.69-1.61) 2:1 (reference) 3:1.22 (0.89-1.69) 4:1.15 (0.83-1.59)	Unadjusted
Li (37) et al., 2017	Inland	Urine iodine concentration (UIC), μg/L 1. <100 μg/L 2.100–199μg/L (reference) 3. 200–299 μg/L 4. >300 μg/L	1:1.76 (0.60-5.15) 2:1 (reference) 3:0.82 (0.21-3.25) 4:2.32 (0.78-6.93)	Unadjusted
Wang (38) et al., 2017	Coastal	Urine iodine concentration (UIC), μg/L 1. <100 μg/L 2.100–199μg/L (reference) 3. 200–299 μg/L 4. >300 μg/L	1:0.93 (0.64-1.35) 2:1 (reference) 3:0.84 (0.59-1.20) 4:1.00 (0.71-1.43)	Unadjusted
Zhang et al. (34), 2016	Inland	Urine iodine concentration (UIC), μg/L 1. <100 μg/L 2.100–199μg/L (reference) 3. 200–299 μg/L 4. >300 μg/L	1: 1.69 ( 0.448-2.554) 2:1 (reference) 3: 0.661 (0.4-1.09) 4:0.85 (0.53-1.36)	Unadjusted
Wang et al. (46), 2024	Coastal	Urine iodine concentration (UIC), μg/L 1. <100 μg/L 2.100–199μg/L (reference) 3. 200–299 μg/L 4. >300 μg/L	1:1.1 (0.8-1.3) 2:1 (reference) 3:0.9 (0.7-1.2) 4:1.1 (0.8-1.4)	Unadjusted

**Table 2:** Differences in the median iodine concentration (in μg/L) in urine between the group without thyroid nodules (TN) and the group with at least one thyroid nodule.

Source	MUIC without TN (μg/L)	N	MUIC with TN (μg/L)	N
Shao et al. (42) 2016	101.5	500	139.4	335
Yuan et al. (43) 2023	121.5	172	164	173
Yan et al. (41) 2021	133.9	1399	123.6	1109
Song et al. (18) 2016	143.1	3716	135.4	1428
Zou et al. (44).2012	143.3	25567	139.5	1385
Tian et al. (40) 2021	167.5	656	154.6	531
Lou et al. (20) 2020	164	420	122.9	2290
Gu et al. (45).2016	165	12035	154	3160
Zhao et al. (39) 2014	169.6	272	184.5	905
Wang et al. (22) 2014	174.3	306	331.3	51

All studies demonstrated adequate participant selection, exposure measurement, blinding of outcome assessment, complete outcome data, and selective outcome reporting. Seven studies adequately addressed and adjusted for confounding variables. Most studies (n=8) adjusted for age and sex in their most adjusted model, while a subset (n=5) also adjusted for BMI.

### Association between iodine status status and thyroid nodules

**Figure 2:**
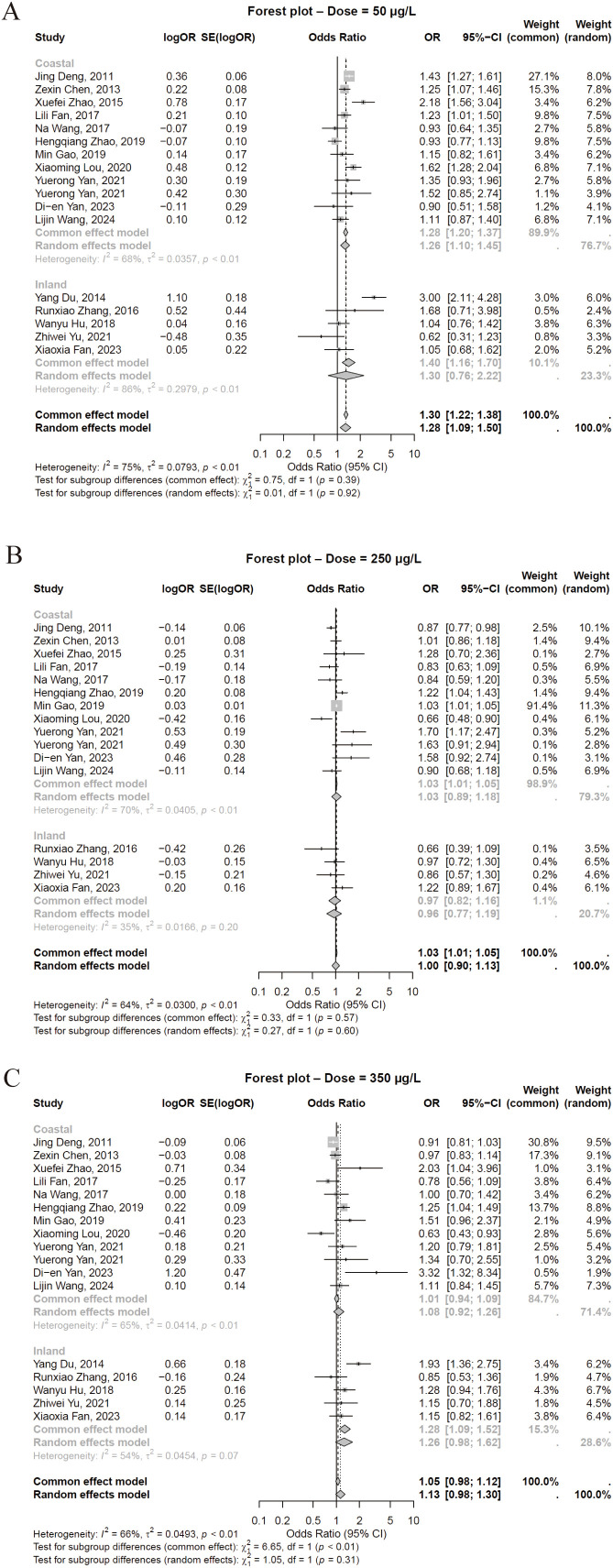
Pooled association between iodine status categories and risk for thyroid nodules. **(A)** Inadequate vs adequate iodine status. **(B)** More than adequate vs adequate iodine status. **(C)** Excessive vs adequate iodine status.

Data on thyroid nodule prevalence across different iodine statusal statuses were extracted from all 18 studies, encompassing 54621 participants. The results of the categorical meta-analysis are shown in **Figure 2**. A comparison between iodine deficiency and adequate status (16 studies) revealed a significantly higher odds of thyroid nodules in the deficiency group (**Figure 2A**, OR = 1.28, 95% CI = 1.09-1.50, *P* < 0.01), with significant between-study heterogeneity (*I*²=75%). The comparison between more-than-adequate and adequate iodine status (15 studies) showed no statistically significant difference in risk (**Figure 2B**, OR = 1.02, 95% CI = 0.93-1.11), with moderate heterogeneity (*I*²=65.0%). Similarly, the comparison between excessive and adequate iodine status (16 studies) indicated no significant increase in nodule risk for the excessive group (**Figure 2C**, OR = 1.13, 95% CI = 0.98-1.30), with moderate heterogeneity (*I*²=66%).

**Figure 3:**
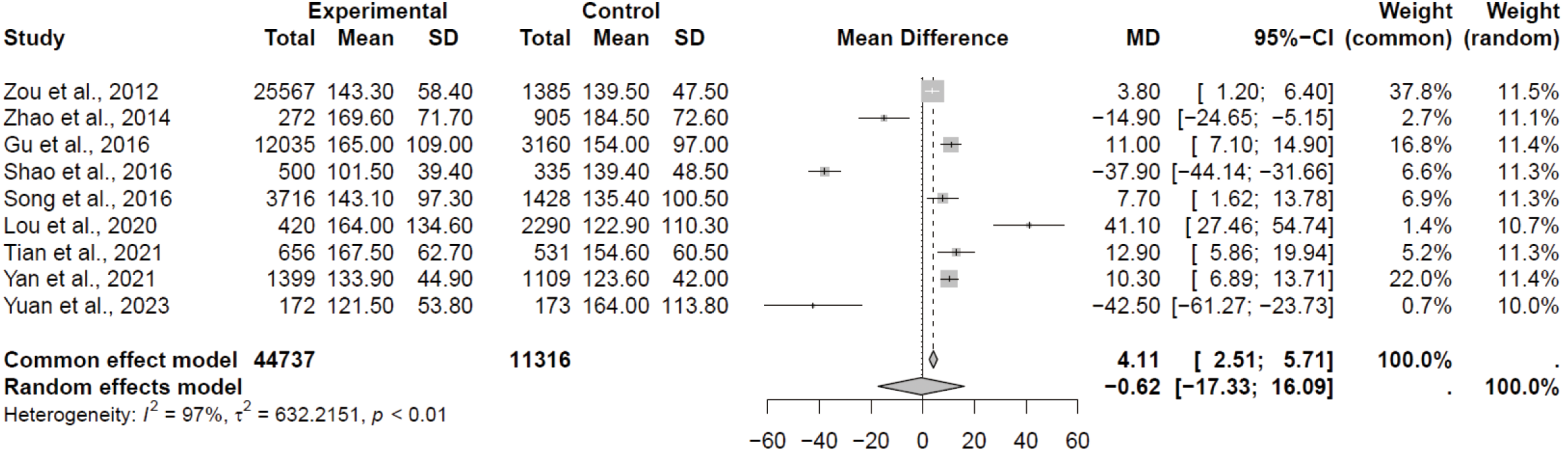
Forest plot showing comparison of difference in MUIC between participants with thyroid nodules and healthy control. MD, mean difference; SD, standard deviation.

**Figure 3** reveals the results of the meta-analysis of the studies with continuous outcomes. Significant heterogeneity was also detected in this case (*I*^2^ = 97.0%), and the pooled results showed no significant association between iodine excessive and risk of having thyroid nodules (MD = -0.62; 95% CI, -17.33 to 16.09). It deserves noting that the extremely high heterogeneity observed across analyses substantially limits the certainty of pooled estimates.

**Figure 4:**
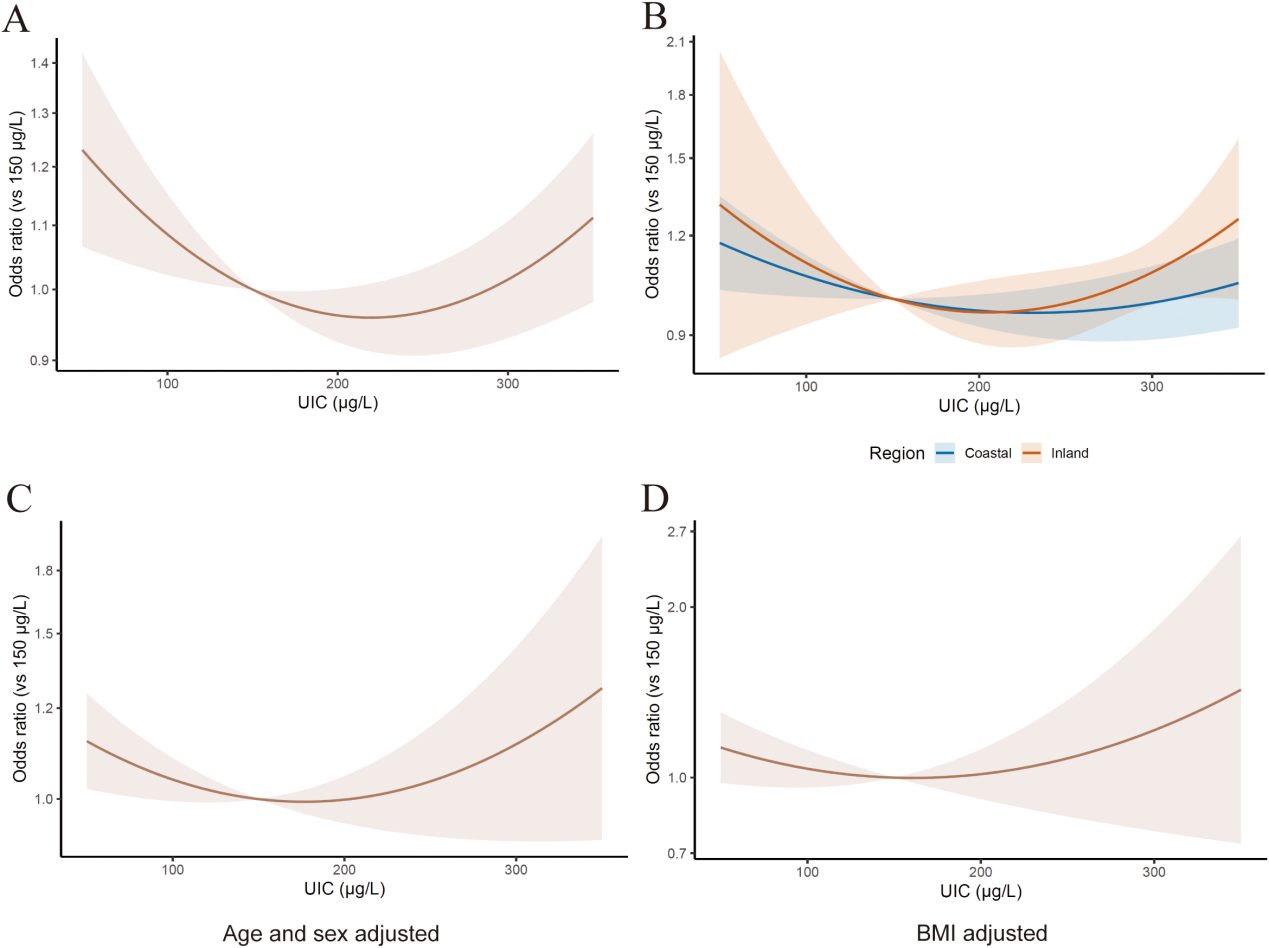
**(A)** Pooled dose-response association between iodine intake and risk for thyroid nodules, estimated using a one-stage approach. The true quadratic relation is presented as a solid line. The relative risks are plotted on the log scale using 150 μg/L as referent. **(B)** Subgroup continuous dose-response analyses plots on association between iodine intake and risk for thyroid nodules with geographical factors stratified (coastal versus inland). **(C, D)** Continuous dose-response analyses plots on association between iodine intake and risk for TNs with sex, age and BMI adjusted.

In the dose-response analysis, we identified a significant nonlinear correlation between UIC and thyroid nodule risk (*P* for nonlinearity < 0.01). The risk was significantly elevated at UIC levels below 100 μg/L or beyond 350μg/L, reaching the lowest risk at 221 (REML)μg/L (**Figure 4A**). Subgroup dose-response analyses with geographical factors stratified (coastal versus inland) (**Figure 4B**) revealed visual differences in magnitude or direction of the correlation between inadequate/excessive iodine intake and the risk of thyroid nodules, although not statistically significant.

### Subgroup and sensitivity analysis

To better characterize the effect of covaries (age, gender, and BMI) on the observed correlations between iodine status status and thyroid nodules risk, we conducted additional sensitivity analyses by pooling ORs from multivariable-adjusted models [4 studies (11, 13–15)]. We observed similar associations between the adequate/excessive iodine intake and thyroid nodules risk using ORs adjusted for age and sex (**Figure 4C**). Additionally, we assessed the role of BMI as a potential contributor to the observed association between iodine status status and thyroid nodules risk through separate pooled analysis using ORs from models that evaluated the association between iodine intake and thyroid nodules risk with adjustment for BMI (**Figure 4D**).

Sensitivity analysis using the leave-one-out method confirmed the robustness of our dose-response findings (**Supplementary Figure S1**). At the iodine deficiency node (50 μg/L), the pooled OR remained significantly elevated (range: 1.24–1.38) after omitting any single study. For the more-than-adequate intake level (250 μg/L), results were generally stable around 1.03, except in one analysis where the estimate became non-significant (OR = 0.98). At the excessive intake node (350 μg/L), the null association persisted (OR range: 1.02–1.12, all 95% CIs included 1). These results indicate that the main conclusions are not driven by any individual study.

### Analysis of heterogeneity, publication bias and evidence certainty

We observed significant heterogeneity across the studies. This heterogeneity was reduced in subgroup analyses and upon exclusion of specific studies in the more-than-adequate (35) and excessive (21) iodine status analyses. Funnel plots for all analysis categories were roughly symmetrical (**Supplementary Figure S2**), *P* value suggesting no statistical evidence of publication bias (*P* = 0.71 and 0.42 for inadequate and more-than-adequate status, respectively). Although *P* value for excessive (*P* = 0.03) status suggested significant publication bias, the funnel plot was roughly symmetrical. The GRADE assessment demonstrated varying levels of certainty across the outcomes (**Supplementary Table S1**). Moderate-certainty evidence supported MUIC differences between participants with/without thyroid nodules. Low-certainty evidence was found for ORs across different iodine statuses.

## Discussion

This systematic review and meta-analysis, which included 54621 participants from 18 cross-sectional studies, identified a nonlinear correlation between iodine status and the risk of thyroid nodules. Both iodine deficiency and excessive iodine intake were correlated with a higher prevalence of thyroid nodules in the included studies; however, causal relationships cannot be inferred due to their cross-sectional design. To our knowledge, this study represents the most comprehensive assessment to date of the dose-response relationship between iodine status and thyroid nodule risk in the general population.

Recent meta-analyses have evaluated the association between USI and thyroid nodules. One such analysis reported a significant rise in the prevalence of thyroid nodules following USI implementation, from 11.0% to 24.4% (39). Similarly, Li et al. noted that while most thyroid disorders remained stable or decreased after a policy adjustment, the prevalence of thyroid nodules alarmingly increased (14). Although not statistically significant in all models, our finding that a more-than-adequate iodine status may be beneficial for nodule prevention is somewhat compatible with another meta-analysis that found the lowest prevalence of thyroid nodules in an excessive-iodine group (UIC >300μg/L) (47). This contrasts with our analysis, which identified the lowest risk in the adequate and more-than-adequate range (UIC = 100-299μg/L). A key limitation of these studies was the lack of a graded dose-response assessment. Our analysis adds to the literature by providing a quantitative, dose-response evaluation that addresses these gaps.

There is a universal consensus on the necessity of adequate iodine intake (UIC = 100-199μg/L, as recommended by the WHO) for thyroid hormone synthesis. However, our dose-response analysis suggests that for the specific prevention of thyroid nodules, the beneficial range of iodine intake may extend beyond this, to 200-299 μg/L, a range also suggested to be beneficial for other thyroid disorders (47). Crucially, regarding iodine consumption exceeding the advised levels (adequate iodine intake status), our study findings suggest that a more than sufficient iodine status status (UIC = 200-299 μg/L, lowest at 221μg/L) is secure and was associated with a lower risk for thyroid nodules. The biological mechanism underlying this nonlinear relationship remains unclear but may involve a U-shaped (hormetic) response. Both deficiency and excess iodine intake exert stress on thyroid follicular cells, while moderate surplus intake maintains homeostasis. It is important to note that the cross-sectional nature of the available evidence limits causal inference. For instance, a study by Yuan et al. proposed that urinary iodine levels are altered in individuals with papillary thyroid cancer, potentially reversing the cause-effect relationship (31). This underscores the need for longitudinal studies and clinical trials to clarify the direction of causality.

Our findings must be interpreted in the context of significant heterogeneity observed across the included studies (*I*² > 90% in some analyses). This high variability likely arises from differences in study populations (e.g., age, sex ratios, geographic location), diagnostic criteria for thyroid nodules, and methods for measuring urinary iodine. While we employed random-effects models and subgroup analyses to account for this, the substantial heterogeneity necessitates caution and indicates that the true relationship between iodine and thyroid nodules is likely modified by other, unmeasured factors. In our subgroup analysis, we observed differing associations between coastal and inland populations, which may partly explain the overall heterogeneity. Coastal residents, who often have diverse dietary iodine sources from seafood, exhibited a different risk profile compared to inland residents, who rely more heavily on iodized salt. This geographical variation in the primary iodine source is a critical effect modifier that should be considered in future research and public health planning.

Globally, the prevalence of thyroid nodules in mainland China mirrors that of other regions with a history of iodine deficiency that have now achieved iodine sufficiency, such as Germany and Mexico (48–51). Overall, the prevalence of thyroid nodules in mainland China mirrored that of other nations and areas with a history of iodine deficiency and current levels of iodine adequacy. Given that 5% of thyroid nodules may be malignant (52), the rising prevalence warrants attention. In response, China has already taken steps to tailor iodized salt concentrations to local needs. Additionally, provincial governments and health administrative departments were granted the authority to establish local standards that fell within 630% of the recommended concentrations based on data for their area (53).

Since the concurrent rise in thyroid nodules occurrences and the mandatory use of iodized salt, an excessive iodine status is deemed a contributing factor to the elevated occurrence of thyroid nodules. Nevertheless, after a synthetic meta-analysis on the occurrence of thyroid nodules across different iodine statusal levels, the prevalence of thyroid nodules decreased initially, and then increased with the rise of iodine intake. Additionally, by synthesizing and comparing the mean UIC between participants with/without thyroid nodules, our findings suggested that those without thyroid nodules generally exhibit higher level of iodine status. Earlier studies indicate that factors such as female gender, diabetes mellitus, substantial betel quid intake, and red meat consumption are independently associated with an increased risk of thyroid nodule formation through multivariate analysis (54). It is essential to consider residual confounding. The concurrent increases in average waist circumference in China following USI implementation are potential confounders, as both are independent risk factors for thyroid nodules. Our analysis could not fully adjust for these factors, and they may contribute to the observed rise in nodule prevalence (40, 55–57).

## Limitations

While this meta-analysis offers a comprehensive overview of the correlation between iodine status and thyroid nodules, several limitations must be acknowledged when interpreting the results. First of all, the predominance of cross-sectional studies in our analysis inherently prevents the inference of causality. The temporal relationship between iodine status and thyroid nodule development cannot be established, as both exposure (UIC) and outcome (nodules) were assessed simultaneously. Secondly, we observed high statistical heterogeneity (*I²* > 90% in some analyses) among the included studies. This heterogeneity likely stems from clinical and methodological variations, such as differences in participant demographics (e.g., age, sex ratio), ultrasound equipment and diagnostic criteria for nodules. The extremely high heterogeneity observed across analyses (*I²* exceeding 90% in several models) substantially limits the certainty of pooled estimates, and the findings should therefore be considered hypothesis-generating rather than definitive. Thirdly, the reliance on spot UIC as a marker of iodine status is a significant limitation. Spot UIC is a useful population-level indicator but is a poor reflection of long-term individual iodine intake due to considerable day-to-day and intra-individual variation. Finally, according to the GRADE framework applied to the body of observational evidence for our primary outcome, the overall certainty of evidence is low. This rating is primarily due to the inherent limitations of the cross-sectional design (risk of bias), substantial unexplained heterogeneity (inconsistency), and the use of spot UIC as an imperfect measure of individual long-term iodine status (indirectness).

## Conclusions

This systematic review and meta-analysis found that more than adequate UIC was likely to be associated with a lower risk for thyroid nodules. Both deficiency and excessive in iodine status are associated with high incidence of thyroid nodules. More-than-adequate iodine intake showed a trend toward a lower prevalence of thyroid nodules. These findings indicate a potential association between iodine status and thyroid nodule risk; however, population-level recommendations regarding salt iodization should be interpreted cautiously and require confirmation from longitudinal and interventional studies.

## Data Availability

The original contributions presented in the study are included in the article/**Supplementary Material**. Further inquiries can be directed to the corresponding author/s.

## Supplementary material

Supplementary Figure S1 Forest plots showing sensitivity analysis results for different iodine status status. **(A)**, sensitivity analysis results for inadequate status (p < 0.01, Tau2 = 0.0795). **(B)**, sensitivity analysis results for more-than-inadequate status (p < 0.01, Tau2 = 0.0296). **(C),** sensitivity analysis results for excessive status (p < 0.17, Tau2 = 0.0494).

Supplementary Figure S2 Funnel plots of the publication bias. A random effects model was used for analysis.
