# Supplementary figures and images for: Correlations between iodine status and the risk of thyroid nodules, a systematic review and dose–response meta-analysis
Source: Front Endocrinol (Lausanne). 2026 Jan 27;17:1711749. doi: 10.3389/fendo.2026.1711749 (PMC12886007; doi:10.3389/fendo.2026.1711749)

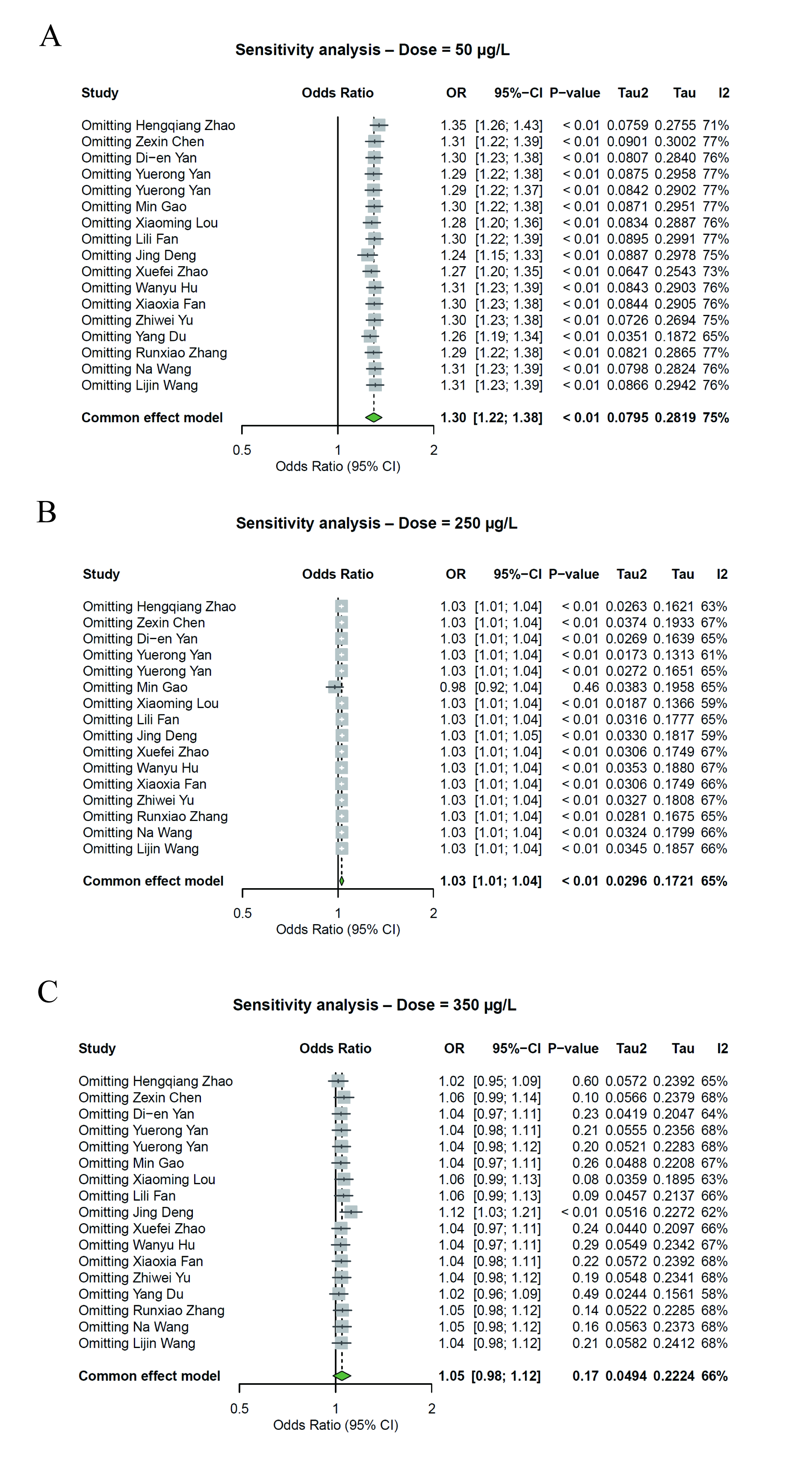

Supplement: Supplementary Figure S1 — Forest plots showing sensitivity analysis results for different iodine status status. (A), sensitivity analysis results for inadequate status (p < 0.01, Tau2 = 0.0795). (B), sensitivity analysis results for more-than-inadequate status (p < 0.01, Tau2 = 0.0296). (C), sensitivity analysis results for excessive status (p < 0.17, Tau2 = 0.0494). [file Image1.tif]

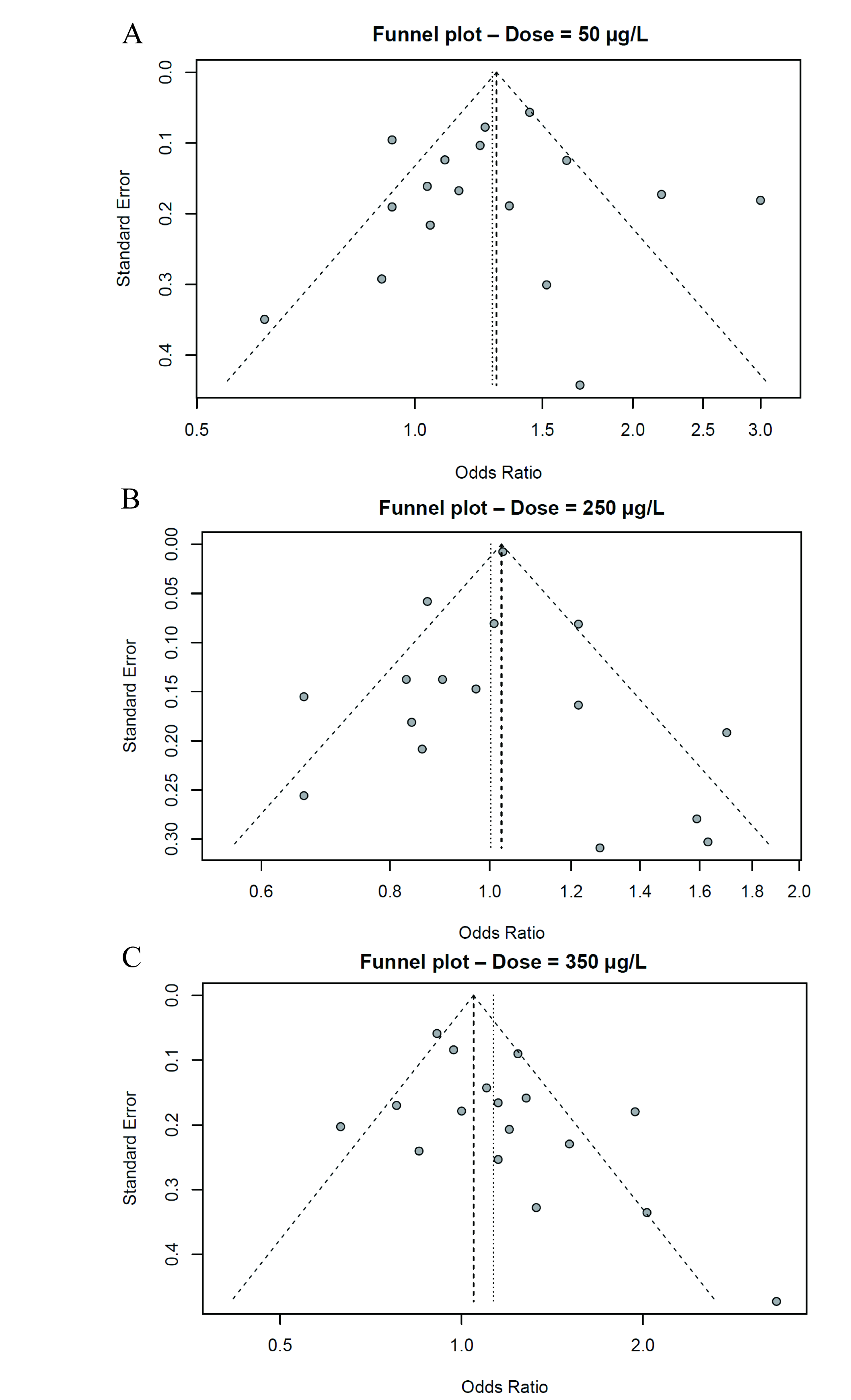

Supplement: Supplementary Figure S2 — Funnel plots of the publication bias. A random effects model was used for analysis. [file Image2.tif]
