# Supplementary material for: Correlations between iodine status and the risk of thyroid nodules, a systematic review and dose–response meta-analysis
Source: Front Endocrinol (Lausanne). 2026 Jan 27;17:1711749. doi: 10.3389/fendo.2026.1711749 (PMC12886007; doi:10.3389/fendo.2026.1711749)
Supplement: Supplementary file 3 [file DataSheet1.docx]

**Supplementary Table S1. GRADE Assessment for outcomes.**

| **Certainty assessment** | | | | | | | **№ of participants** | | **Effect** | | **Certainty** |
| --- | --- | --- | --- | --- | --- | --- | --- | --- | --- | --- | --- |
| **№ of studies** | **Study design** | **Risk of bias** | **Inconsistency** | **Indirectness** | **Imprecision** | **Other considerations** | **Experimental (cases)** | **Control (cases)** | **Relative (95% CI)** | **Absolute (95% CI)** |  |
| **Iodine status** |  |  |  |  |  |  | **UIC, <100 μg/L** | **UIC, 100–199 μg/L** |  |  |  |
| 16 | Cross-sectional studies | not serious | not serious | serious^a^ | serious^a^ | publication bias strongly suspected^b^ | 12659(3399) | 18777(4546) | OR **1.28** (1.09 to 1.1.50) |  | ◯◯⨁⨁ Low^a,b^ |
| **Iodine status** |  |  |  |  |  |  | **UIC, 200–299 μg/L** | **UIC, 100–199 μg/L** |  |  |  |
| 15 | Cross-sectional studies | not serious | not serious | serious^a^ | serious^a^ | publication bias strongly suspected^b^ | 10409(2335) | 18777(4546) | OR **1.03** (0.90 to 1.13) |  | ◯◯⨁⨁ Low^a,b^ |
| **Iodine status** |  |  |  |  |  |  | **UIC, >300 μg/L** | **UIC, 100–199 μg/L** |  |  |  |
| 16 | Cross-sectional studies | not serious | not serious | serious^a^ | serious^a^ | publication bias strongly suspected^b^ | 8663(1970) | 18777(4546) | OR **1.05** (0.98 to 1.30) |  | ◯◯⨁⨁ Low^a,b^ |
| **MUIC, μg/L** |  |  |  |  |  |  | **With nodules** | **Healthy control** |  |  |  |
| 9 | Cross-sectional studies | not serious | not serious | serious^a^ | not serious^a^ | publication bias strongly suspected^b^ | 11367 | 45043 | - | MD **4.11** (2.51 to 5.71) | ◯⨁⨁⨁ Moderate^a,b^ |
|  | | | | | | |  | | | | |

**CI:** confidence interval; **MD:** mean difference

**Explanations**

a. Downgrade one level for the inconsistency because of the heterogeneity.

b. Downgrade one level for the other considerations because of the substantial publication bias.
